# Intimate partner violence disclosure and associated factors among pregnant women attending a city hospital in South-Western Uganda: a cross-sectional study

**DOI:** 10.1186/s12884-022-04812-x

**Published:** 2022-06-13

**Authors:** Eve Katushabe, John Baptist Asiimwe, Vincent Batwala

**Affiliations:** 1 Department of Community Health, Mbarara University of Science and Technology, P.O. Box 1410, Mbarara, Uganda; 2 Faculty of Nursing and Health Sciences, Bishop Stuart University, P. O. Box 09, Mbarara, Uganda; 3 School of Nursing and Midwifery, Aga Khan University, Uganda Campus, Kampala, Uganda; 4 Directorate of Research and Graduate Training, Mbarara University of Science and Technology, P. O.Box 1410, Mbarara, Uganda

**Keywords:** Intimate partner Violence Disclosure, Pregnancy, Choice of disclosure, Uganda

## Abstract

**Background:**

Intimate partner violence (IPV) during pregnancy is a public health problem in Uganda that negatively impacts maternal and newborn health outcomes. However, IPVdisclosure and associated factors among pregnant women have remained poorly documented in southwestern Uganda. Therefore, this study determined IPV disclosure and associated factors among pregnant women attending a large City hospital.

**Methods:**

In a cross-sectional design, 283 women attending Mbarara City Hospital Antenatal care (ANC) clinic were consecutively recruited into the study. Data was collected using a semi-structured questionnaire. This was administered by the research team and the exercise took over a month. That is; from 7^th^ January 2019 to 7^th^ February 2019. The collected data was entered in STATA, and it was analyzed using chi-square, and univariate logistic regression statistics.

**Results:**

Out of the 283 pregnant women who participated in the study, 199 of them, representing seventy-point three percent (70.3%), had reportedly experienced at least one type of IPV during their current pregnancy. However, nearly fifty percent of those that experienced IPV (49.7%, *n* = 99) disclosed it to a third party, while the majority disclosed it to their biological family member (66.7%), followed by their friends (55.5%), members of their husband’s family (35.3%), neighbors (12.1%), healthcare providers (9.1%), religious leaders (8.1%), and the police (3.1%). Gravidity, OR = 1.9(95% CI: 1.07–3.31, *p* = 0.027), parity OR = 1.9(95% CI: 1.08–3.34, *p* = 0.026) and witnessed IPV OR: 5.4(95% CI: 1.93–14.96; *p* = 0.001) were significantly associated with IPV disclosure.

**Conclusion:**

A large proportion of the pregnant women who experienced IPV did not disclose it to any third party. In addition to the above, pregnant women's characteristics seem to have a strong influence on IPV disclosure. Therefore, it is important for healthcare providers to routinely screen for IPV during antenatal care if a high IPV disclosure rate is to be achieved.

## Background

Intimate Partner Violence (IPV) during pregnancy is a significant public health problem worldwide [1], affecting about 30% of pregnant women aged 15 years and above [2]. In sub-Saharan Africa, the prevalence of IPV varies from one country to another. It ranges from 2 to 57%, [3, 4], with the East African region accounting for 39% of the IPV burden [5]. In eastern Uganda, different forms of IPV during pregnancy were reported to be at 27.8% [6]. Above all, six out of 10 women were reported to suffer at least one type of IPV in their lifetime, in Uganda (UBOS, 2017).The process of IPV disclosure if appropriately conducted, can be an effective strategy to cope with the violence [7].That process may end IPV which may as a result, guarantee the wellbeing of the mother, her pregnancy, as well as, help in the formulation of strategies for the prevention of future occurrences of IPV [8]. Failure to disclose may expose pregnant women to maternal mental health problems [9], reduced maternal weight, increased likelihood of undergoing cesarean section delivery, maternal mortality [10, 11], and inadequate uptake of ANC [12]. Hence failure to disclose becomes an obstacle to the achievement of the safe motherhood initiative [13]. Fetal effects of failure to disclose include premature birth and intrauterine fetal demise [10, 11]. Notwithstanding the above effects of failure to disclose, IPVdisclosure remains low among pregnant women in general. For instance, among pregnant women in Nigeria (28.6%) [14] and Tanzania (23.3%) [15], lower proportions of pregnant women disclosed IPV experience to a third party.

According to the previous literature, there are multiple factors associated with the low IPV disclosure rate among prenatal women and these include unemployment, unplanned pregnancy, lack of trust in the health care professionals and the insufficient time given to these women during ANC visits [15]. However the main reasons for failure to disclose IPV among women in general, include fear of the perpetrator, feeling uncomfortable with the health care providers and the feeling that IPV was not serious [16].

ANC contacts provide an opportunity for disclosure and intervention that could reduce the adverse effects of IPV during the perinatal period [17]. Most pregnant women in developing countries interact with healthcare workers during the ANC period. In Uganda, the ANC policy recommends at least eight visits during pregnancy with a likelihood of continued monitoring, providing a perfect opportunity for reporting and discussing IPV [4].

However, there is little research evidence around IPV disclosure among pregnant women attending ANC in Uganda. In this setting, earlier IPV studies reporting IPV experiences only focused on the general population, while few investigated IPV prevalence in pregnancy [6, 18, 19]. Therefore, this study determined IPV disclosure and associated factors among pregnant women attending a large City hospital.

## Methods

### Study design, setting, and population

This study employed a cross-sectional design. The study was conducted among pregnant women attending the ANC clinic of Mbarara City Hospital in southwestern Uganda during the month of January 2019 (4 weeks). The hospital operates a daily general outpatient, ANC, family planning and young child clinics; and an in-patient maternity ward. In 2018, the hospital ANC register indicated that about 800 pregnant women attended ANC monthly (new ANC cases and re-attendance) and resided in and outside Mbarara City.

### Participants

**Fig. 1:**
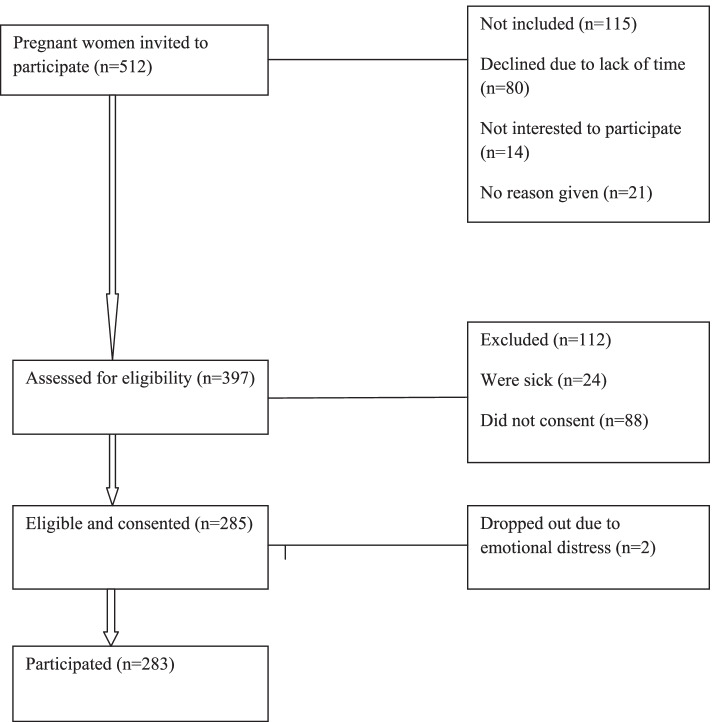
The flow diagram for the study participants

Women aged 15 years and above, with a confirmed diagnosis of pregnancy (ultrasound results), at any gestation age attending antenatal checkups, were not sickly and had consented were included in the study (Fig. 1). In all, 397 were assessed for eligibility, 285 were eligible and consented, 2 pregnant women dropped out due to emotional distress, 283 participated and were screened for recent IPV experience during the current pregnancy.

### Sample size and sampling

A sample of 273 pregnant women was determined for recruitment using a standard formula by Kish [20], where we assumed the IPV disclosure rate of 23.3% as reported in a study conducted in Tanzania [7] and the probability (*p*-value) was set at 0.05. The participants were consecutively sampled as they left the hospital ANC clinic after receiving ANC services.

### Data collection procedure

Data collection was carried out over a period of one month from 7^th^ January 2019 to 7^th^ February 2019. After obtaining ethical approval from Mbarara University research ethics committee and administrative clearance from Mbarara city town clerk and Mbarara City Hospital managers, the principal investigator (Midwife) recruited three (3) research assistants (baccalaureate nursing students in their final year) and trained them to collect data. After the training, the study tools were pre-tested on ten (10) pregnant women at Mbarara Regional Referral Hospital, after which adjustments to the tools were made. The research team then briefed the ANC clinic staff (midwives), and the probable participants during the general health education sessions (held daily except on Thursdays) about the study objectives/purpose and the data collection procedure.

The research team later individually contacted and reminded probable participants about participating in the study, as they exited the ANC clinic, and those that accepted were linked to the private consultation rooms in the ANC clinic and screened against the eligibility criteria. Those participants that met the inclusion criteria were provided with full information about the study in their native language (Runyakore/Rukiga), using an information sheet. Participants later signed the consent form that was read to them by the researcher, and the uneducated confirmed consent with a thumbprint.

The research team interviewed the study participants in the local language using a semi-structured questionnaire. Each interview took between 15 and 30 min. The filled questionnaires were manually checked for completeness before leaving the participants. During the interview, participants were given refreshments (energy drink and a cake). After the interview, the study participants were thanked for their participation in the study.

## Study variables and measures

### Outcome measures

#### Presence of intimate partner violence

Data was collected using the World Health Organization (WHO) study questionnaire for assessing IPV among women [21] which has been used in IPV studies in sub-Saharan Africa [14, 22]. This questionnaire was found to be effective in distinguishing between the three types of IPV among pregnant women [23]. The presence of IPV was assessed during the current pregnancy and was defined as participants who experienced one of the three types of IPV, namely: psychological, physical and sexual violence.

The answer options were “Yes”, and “No”. Participants who experienced psychological IPV were indicated as those who responded “Yes” to; restrictions from seeing friends and family members of origin by their sexual partner, intimidated on purpose, demeaned before others; and threatened to be injured. Participants who responded “Yes” to: beaten up or punched, strangled, and threatened or attacked with a gun/knife or any other weapon were said to have experienced physical IPV. Participants who responded with “Yes” to; had involuntary sex with their partners, as a result of fear of what the spouse would do or had sex in a way that was humiliating were said to have experienced sexual IPV.

#### IPV disclosure

The primary outcome of this study was IPV disclosure measured as a binary variable (Yes/No). Participants that mentioned “Yes” were considered to have disclosed IPV to a third party, and vice versa if a participant said “No”. Participants were also asked to mention the person they disclosed IPV experience to and why.

### Independent variables

#### Socio-demographic and clinical characteristics:

Items measuring socio-demographic characteristics were constructed from the literature. This included age, level of education, occupation, tribe, marital status, reproductive history such as how many pregnancies they have had including the current (gravidity), how many children they have given birth to (parity), was the current pregnancy intended (pregnancy intention) and hazardous alcohol use (AUDIT-C score with cut off of ≥ 3 indicating hazardous alcohol use) [24, 25].

#### Social and situational factors

Since communication is a key factor in the disclosure of IPV, one item (1) was constructed to measure the frequency of communication of the study participants to their family members or friends [15]. The frequency was measured in terms of either once a week, a month, a year or more. In addition one (1) item was constructed to measure the factors that surrounded the IPV event that may have had an effect on IPV disclosure such as the presence of witnesses or third parties [26].

### Data analysis

The filled questionnaires were cleaned before data entry in EpiData 3.1 software (The EpiData Association, Odense, Denmark) and analyzed in STATA (v.14, Stata Corp. LP, College Station, Texas, USA). All open-ended questions in the questionnaire were coded before entry. Univariate analysis was carried out to describe the background characteristics of the participants using frequencies and percentages. A normality test was conducted for continuous variables. Variables that were not normally distributed, their medians, and ranges are reported. Bivariate analysis using the Chi-square test or Fisher's exact test statistics was performed to determine the association between independent variables and IPV disclosure. The probability value (*p*-value) was set at the 0.05 level of significance, and the confidence intervals (CIs) were calculated at the 95% level. To identify the independent predictors of IPV disclosure, a univariate logistic regression was conducted and unadjusted Odds Ratios (uOR) reported.

### Ethical consideration

Ethical approval was obtained from the Faculty of Medicine Research Committee (DMS 6 # 12/09–18) and the Mbarara University of Science and Technology Research Ethics Committee (MUREC # 22/09–18). In addition, the study followed the ethics and safety recommendations for research on IPV [27]. Prior to the study, the midwives at the Antenatal care clinic and the research team members received a brochure with an overview of services for women and families experiencing IPV including referral to community services such as legal aid, police, child welfare services, sexual assault services, advocacy and support. All participating pregnant women received a card with a list of phone numbers to call in case they did not feel safe. All the study participants provided consent in writing or through their thumbprint after being informed about the study. Interviews were paused for participants who suffered emotional distress (*n* = 03), but later were resumed after the participants had recollected themselves. Participants who failed to re-collect (*n* = 02) were referred to the study counselor for continuous support. Confidentiality was protected throughout data collection to ensure women’s safety and data quality.

## Results

### Socio-demographic and clinical characteristics of pregnant women

Overall, 283 ANC women who attended Mbarara City Hospital during January 2019 were recruited into the study (Table 1). Approximately fifty percent (50.2%) of the participants were aged 20–24 years, with the youngest and eldest being 15 and 49 years old respectively. A similar proportion of participants (50.2%) were in the second trimester of their current pregnancy and prime gravidas (51.6%). Nearly fifty-five percent (54.8%) of the pregnant women had never had any child and this included the first pregnancy and those that had miscarriages. The majority (93.6%) were living with their sexual partners. Only seventy-one percent (71%) intended to conceive the current pregnancy. Most of the participants were Anglicans (50.2%) by religion, followed by Catholics (38.9%), Muslims (9.5%), and others. The Banyankore ethnic group constituted the majority of the participants (72.8%). Regarding employment, forty-one percent (41%) of the participants were self-employed. Nearly forty-five percent (44.5%) of the participants had attained secondary education whereas the majority of the study participants (92.9%) were not alcohol users.

**Table 1:** Socio-demographic and clinical characteristics of study participants

Variable	*n (%)*
**Age in years**	**24(16–25)**^a^
15 – 19	23(8.1)
20 – 24	142(50.2)
25–29	83(29.3)
30–34	29(10.3)
≥ 35	6(2.2)
**Trimester at time of interview**	
1^st^	15(5.3)
2^nd^	142(50.2
3^rd^	126(44.5)
**Gravidity**	**1(1–5)**^a^
1	146 (51.6)
2	71(25.1)
3	46(16.3)
≥ 4	20(7)
**Parity**	**0(0–5)**^a^
None	155(54.8)
1	69(24.4)
2	38(13.4)
3	13(4.6)
4	7(2.4)
≥ 5	1(0.4)
**Intended pregnancy**	
Yes	201(71)
No	82(28.9)
**Religion**	
Anglican	140(50.2)
Catholic	109(38.9)
Muslim	27(9.5)
Seventh Day Adventist	4(1.4)
Others	3(1.1)
**Tribe**	
Munyankore	206(72.8)
Mukiga	39(13.8)
Muganda	24(8.5)
Others	14(5)
**Marital status**	
Living with a partner	265(93.6)
Separated	14(5)
Single	4(1.4)
**Occupation**	
Salaried job	67(23.7)
Self-employed	116(41)
Not employed	100(35.3)
**Education level**	
No formal education	93(1.1)
Primary	62(21.9)
Secondary	126(44.5)
Tertiary	92(32.5)
**Hazardous alcohol use**	
Yes	20(7.1)
No	263(92.9)

^a^Median (Range)

### The IPV prevalence

Out of the 283 pregnant women enrolled, 199 (70.3%) had experienced some form of IPV in their current pregnancy (Table 2). Psychological IPV was the most prevalent (38.2%). None of them had experienced exclusively physical violence. The majority of the study participants had experienced psychological and sexual IPV (22.3%) whereas about four percent (3.5%) had experienced all three types of IPV.

**Table 2:** Prevalence and forms of IPVamong pregnant women

Variable (*N* = 283)	*n (%)*
Experienced IPV	199(70.3)
**Forms of IPV**	
**One**	
Psychological	108 (38.2)
Sexual violence	9(3.2)
Physical violence	0(0)
**Two**	
Psychological and sexual	63(22.3)
Psychological and physical	8(2.8)
Physical and pexual	1(0.4)
**Three**	
Psychological, physical, and sexual	10(3.5)

### The Prevalence of IPV Disclosure

Out of the 199 women who experienced violence in their current pregnancy, about fifty percent of them (49.7%) disclosed it to a third party (Table 3). Most of the participants informed their biological family members (66.7%) and only nine-point one percent (9.1%) of the participants disclosed it to the healthcare providers.

**Table 3:** The prevalence of IPV disclosure

Variable	Disclosure	
	Yes *n (%)*	No *n (%)*
**Overall**	99 (49.7)	100(50.3)
**Person of disclosure**^a^		
Health worker	9(9.1)	90(90.9)
Husband’s family of birth	35(35.3)	64(64.6)
Woman’s family of origin	66(66.7)	33(33.3)
Neighbor	12(12.1)	87(87.9)
Religious leader	3 (3)	96(97)
Woman’s friend(s)	51(55.5)	48(48.5)
Police	8 (8.1)	91(91.9)
Others	3 (3)	96(97)

^a^Multiple response questions

### Reasons for the IPV experience disclosure

The majority of the participants disclosed IPV because they wanted to access support from those they disclosed to (96.5%, Table 4). On the other hand, fewer participants (15.2%) disclosed their IPV experience after observing their children suffering.

**Table 4:** Reasons for the disclosure of IPV experience among pregnant women

**Reason (s) for IPV disclosure**	*n* (%)^a^
Access support	108 (96.5)
Respect for women’s needs and wishes	82 (73.2)
Personal safety	78 (69.6)
Could not endure anymore	76 (67.9)
Keeping other family members/loved ones safe	39 (34.8)
Threatened or tried to be killed	25 (22.3)
Observed children were suffering	17(15.2)

^a^Multiple responses

### Factors associated with IPV disclosure among pregnant women

In bivariate analysis, findings indicate that only gravidity (*p* = 0.027), parity (*p* = 0.026), and witnessing of IPV by a third party (*p* = 0.001) were significantly associated with IPV disclosure (Table 5). In univariate logistic regression, experiencing IPV in the presence of a third party was the most important factor that influenced IPV disclosure. Pregnant women who had experienced IPV in the presence of a third party were about five times more likely to disclose to other third parties compared to those who had experienced IPV with no one present (OR = 5.7, 95%CI: 2.09–15.83, *p* = 0.001). Multigravidas (who had carried two or more pregnancies) were 1.9 times more likely to disclose IPV to a third party than those carrying their first pregnancy (OR = 1.9, 95% CI: 1.07–3.31, *p* = 0.027). Likewise, multiparous women with one or more children were 1.9 times more likely to disclose IPV to a third party than the nulliparous women (OR = 1.9, 95% CI: 1.08–3.34, *p* = 0.026).

**Table 5:** Bivariate analysis of factors influencing IPV disclosure among pregnant women

Variable		Disclosure		UOR (95%CI)	*p*-value
***N* = 199**		Yes, *n (%)*	No, *n (%)*		
**Age (years)**	15–29	84(48)	91(52)	1.0	0.180
	30 +	15(62.5%)	9(37.5%)	1.8(0.75–4.34)	
**Trimester at time of interview**	1^st^	4(40)	6(60)	1.0	0.799
	2^nd^	48(49.5)	49(50.5)	1.5(0.39–5.54)	
	3^rd^	47(51.1)	45(48.9)	1.6(0.41- 5.92)	
**Gravity**	First pregnancy	46 (42.9)	61(57.0)	1.0	0.027*
	≥ 2 pregnancies	54(58.7)	38(41.3)	1.9(1.07–3.31)	
**Parity**	None	48 (43.2)	63(56.8)	1.0	0.026*
	≥ 1	52(59.1)	36(40.9)	1.9(1.08–3.34)	
**Intended Pregnancy**	Yes	63(46.7)	72(53.3)	1.0	0.207
	No	36(56.3)	28(43.8)	0.7(0.37–1.24)	
**Religion**	Catholics	43(57.3)	32	1.0	0.206
	Anglican	47(48)	51(52)	0.7(0.37–1.26)	
	Others	10(38.5)	16(61.5)	0.5(0.19- 1.16)	
**Tribe**	Munyankore	75(52.5)	68(47.6)	1.0	0.322
	Others	25(44.6)	31(55.4)	0.7(0.39–1.36)	
**Marital status**	Living with partner	92(49.5)	94(50.5)	1.0	0.4
	Not living with a partner	8(61.5)	5(38.5)	1.1(0.60- 1.91)	
**Occupation**	Gainfully employed	63(49.6)	64(50.4)	1.0	0.809
	Not employed	37(51.4)	35(48.6)	1.1(0.60–1.91)	
**Education level**	None or primary education	25(52.0)	24(49)	1.0	0.901
	Secondary and above	45(51.1)	43(48.9%)	0.9(0.50–1.83)	
**Communication to the family of birth/partner**	At least once a week	86(51.5)	81(48.5)	1.0	0.422
	Once a month and above	14(43.8)	18(56.3)	0.7(0.34–1.57)	
**Forms of violence**	One form	67(57.3)	50(42.7)	1.0	0.061
	Two forms	29(40.3)	43(59.7)	1.9(1.05–3.48)	
	Three forms	4(40)	6(60)	1.9(0.52–7.24)	
**Witnessed IPV**	No	77(45)	94(55)	1.0	0.001*
	Yes	23(82.1)	5(17.9)	5.7(2.03–15.46)	

^*^Statistically significant

## Discussion

This study investigated the prevalence of Intimate Partner Violence disclosure and the associated factors among pregnant women attending a large hospital situated in an urban setting in southwestern Uganda. The study’s findings indicate that a large proportion of the study participants had experienced at least one type of IPV (70.3%) before the study but during the current pregnancy among them only about fifty percent of them (49.7%) had disclosed their IPV experience to a third party.

However, this study proportion of IPV disclosure was higher than that reported in Tanzania (23.3%) [7], Nigeria (46%) [28], and Dhaka (21%) [29], but similar to the one reported in Ethiopia (51.4%) [30]. Although the reason for the difference in IPV disclosure rate between Uganda and other countries is unclear, it appears to be related to cultural barriers. For example, a study in Tanzania described IPV exposure as a normal event, as a result, IPV disclosure caused embarrassment to victims of the violence [31].

The findings of this study also indicated that the majority of the pregnant women (66.7%) had disclosed IPV to their biological family members. These results are comparable with a Nigerian study wherein, an equivalent proportion of women (68%) expressed the readiness of IPV disclosure to the kinsfolk [28]. The probable reason for IPV victims to prefer disclosing to their biological family members might be because of the solid personal connection between them, unlike other members of the community. In addition, the victim's in-laws and friends of the violent intimate partner were found to be less supportive of the IPV victims [32]. Other studies have associated the fear of revenge, fear of getting into trouble with the perpetrator, the feeling that the situation was not worth reporting, and keeping the IPV event more private with IPV non-disclosure [14].

Surprisingly, fewer participants of the study (9.1%) disclosed their IPV experience to healthcare providers. This percentage is lower than that reported in Serbia (25.7%) [33], but it is unacceptably low considering that pregnancy increases women’s contact with health care staff particularly midwives who provide valuable information to benefit the mother and her fetus. These pregnant women experiencing IPV need counseling services because of the adverse effects on the fetus and the mother. If disclosure to the health care provider’s increases, then IPV-associated complications would be reduced.

However, the major reasons for the failure of pregnant women from disclosing IPV to the health workers included; feeling uncomfortable with the health care providers [16], perceived absence of privacy, unsuitable means of probing, and stigmatizing attitude from care providers [17, 21]. The lack of trust in service providers and insufficient time in talking over IPV with ANC clients contributed to the failure of disclosure to health providers by pregnant women [15].

The current study findings revealed that women who experienced violence in the presence of a third party were more likely to disclose IPV experience to other third parties similar to findings of a study conducted in the United States of America [26]. The IPV witnesses may provide courage, confidence, and guidance for the victim to seek support elsewhere. Previous research reported that motivation for IPV disclosure was having children in a violent relationship witnessing IPV [21, 29, 30]. This could be attributed to the women’s fear of the effect of IPV on children since they might also be threatened or harmed by the perpetrator. Overall, this study seems to suggest that pregnant women's characteristics have a strong influence on IPV disclosure.

## Recommendations

Currently, in Uganda, there are no recommended strategies targeting the identification and management of IPV in clinical settings. The current essential maternal and newborn clinical care guidelines (2021), require health care providers to screen for IPV throughout the 8 ANC visits.

However, the above guidelines are silent on how the screening should occur and the subsequent management of the victims of IPV. Therefore, a detailed policy on screening and management of IPV should be incorporated in the clinical guidelines.

In addition to the training of midwives on how to identify and manage IPV cases, and given the huge workload on the part of the healthcare staff, the policy should incorporate or advocate for the recruitment of a counselor, who would assist the midwives in the management of IPV cases. There is also a need to develop a customized tool to measure IPV in the African setting.

## Limitation

Since IPV is a culturally sensitive issue in Uganda, there is a possibility that participants provided socially desired responses. However, this was minimized by ensuring anonymity, confidentiality, and training of the data collectors on how to handle the data collection process.

Secondly, because of the low sample size included in this study, we were unable to conduct multivariate analysis, which would have controlled for confounding variables thus providing reliable predictors.

In addition, the current study did not capture the economic violence type of IPV, which may have altered significantly the findings of this study. Therefore, further studies should include economic violence type of IPV.

## Conclusion

The IPV burden in this clinical setting is very high and widespread among pregnant women. However, about half of them disclosed their IPV experience. Pregnant women preferred to disclose IPV to their biological family members and less to health care providers. Additionally, pregnant women's characteristics seem to have a strong influence on IPV disclosure. Therefore, it is important for health care providers to routinely screen for IPV during antenatal care if a high IPV disclosure rate is to be achieved.

## Data Availability

The dataset used in this research is available from the corresponding author on reasonable request.

## Abbreviations

ANC: Antenatal Care
IPV: Intimate Partner Violence
WHO: World Health Organization
